# Personality characteristics, defense styles, borderline symptoms, and non-suicidal self-injury in first-episode major depressive disorder

**DOI:** 10.3389/fpsyg.2023.989711

**Published:** 2023-01-26

**Authors:** Bo Peng, Jiwu Liao, Yang Li, Guangbo Jia, Jihui Yang, Zhiwei Wu, Jian Zhang, Yingjia Yang, Xinxin Luo, Yao Wang, Yingli Zhang, Jiyang Pan

**Affiliations:** ^1^Department of Psychiatry, The First Affiliated Hospital of Jinan University, Guangzhou, Guangdong, China; ^2^Department of Depressive Disorder, Shenzhen Kangning Hospital, Shenzhen, Guangdong, China; ^3^Shenzhen Mental Health Center, Shenzhen, Guangdong, China; ^4^Teachers College, Columbia University, New York, NY, United States

**Keywords:** non-suicidal self-injury, depression, personality, traits, defensive mechanisms, borderline

## Abstract

**Background:**

Non-suicidal self-injury (NSSI) is commonly seen in adolescents with depression and is a high-risk factor leading to suicide. The psychological mechanisms underlying depression with NSSI are still unclear. The purpose of this study was to explore the differences in personality traits, defensive styles, and borderline symptoms among first-episode youth patients with depression and self-injury compared with patients with depression without self-injury and healthy populations.

**Methods:**

The current study recruited 188 participants, including 64 patients with depression and NSSI, 60 patients with depression without NSSI, and 64 healthy control subjects. Eysenck Personality Questionnaire, the Defense Style Questionnaire, the short version of the Borderline Symptom List, the Beck Depression Inventory, and the Ottawa Self-Injury Inventory were used to assess all participants.

**Results:**

Patients with depression and NSSI showed more psychoticism than patients with depression without NSSI and healthy control subjects. Patients with depression and NSSI presented more intermediate defense styles than healthy control subjects. In the patients with depression and NSSI group, the frequency of self-injury in the last week was negatively correlated with mature defense styles and positively correlated with depressive symptoms and borderline symptoms. Further regression analysis showed that EPQ-psychoticism and depressive symptoms were independent risk factors for NSSI in patients with depression.

**Conclusion:**

This study found that patients with depression and self-injury presented more neuroticism, introversion, EPQ-psychoticism, immature defenses, intermediate defenses, and borderline symptoms. Self-injury frequency was negatively correlated with mature defense styles and positively correlated with depressive symptoms and borderline symptoms. EPQ-Psychoticism and depressive symptoms are risk factors for predicting non-suicidal self-injury in patients with depression.

## 1. Introduction

Non-suicidal self-injury (NSSI) refers to any intentional behavior by an individual without suicidal intent that results in immediate destruction of bodily tissue and is not socially acceptable (Lloyd-Richardson et al., 2007). The American Psychiatric Association included NSSI as Disorders Requiring Further Study in Section III of the DSM-5 and assigned corresponding diagnostic criteria (American Psychiatric Association D, Association AP, 2013). NSSI is a common mental health threat among adolescents, most often seen in mid-adolescence and often first occurring in early adolescence (Plener et al., 2015), and has become one of the major health challenges for adolescents worldwide. A recent meta-analysis showed a lifetime prevalence of NSSI was 16.9% (95% CI 15.1–18.9) in nonclinical individuals (Gillies et al., 2018), and 12.2–22.37% in the Chinese adolescent population (Lang and Yao, 2018; Tang et al., 2018). NSSI is a risk factor (Gillies et al., 2018) and a strong predictor of individuals taking suicidal behavior (Scott et al., 2015; Law and Shek, 2016). In recent years, an increasing number of studies have found that NSSI is common in major depressive disorder patients (MDD; Andover, 2014; Ose et al., 2021), especially in adolescents and young adults with depression (Xiao et al., 2020). Longitudinal studies suggest that depression is the most likely risk factor for NSSI (Plener et al., 2015; Lim et al., 2020). NSSI may aggravate the depression condition and even increase patients’ suicide risk by more than seven times (Ose et al., 2021), suggesting that the interventions for NSSI can prevent and reduce suicide in adolescents with depression. Therefore, understanding the pathological mechanisms of depression with NSSI is very crucial for suicide prevention. Research on the pathological mechanisms of depression with NSSI is still unclear, but some studies have found that having difficulties in emotion regulation, alexithymia, and childhood trauma may be risk factors for NSSI in adolescents with depression (Taş Torun et al., 2021; Bordalo and Carvalho, 2022). Our previous study has also found that patients with depression and NSSI have a dysfunctional HPA axis (Peng et al., 2022). Patients with depression and NSSI responded worse to antidepressant medication than those without NSSI (Forbes et al., 2020). Therefore, it can be seen that there may be differences in pathological mechanisms between MDD with or without NSSI, but there are very few studies focusing on the differences in personality factors between the two.

Psychological factors, especially the personality traits, play a very important role in the occurrence, development, and prognosis of psychiatric disorders (Franquillo et al., 2021). Research of personality traits in psychiatric disorders facilitates prevention and targeted treatment measures. Neuroticism, EPQ-psychoticism, and extroversion are three important dimensions of personality, and many studies suggest that neuroticism is closely related to a wide range of psychological problems and a variety of psychiatric disorders (Khan et al., 2005; Malouff et al., 2005). Among these three important dimensions of personality, EPQ-psychoticism is highly correlated with the antagonism/agreeableness dimensions (The disagreeable/antagonistic individual is egocentric, suspicious of others’ intentions, and competitive rather than co-operative) of the big five personality (McCrae and John, 1992). Hsu and Chen (2013) found that psychiatric outpatients with NSSI presented higher levels of introversion and neuroticism than healthy individuals. Another large sample study from the United Kingdom, Hafferty et al. (2019) found that neuroticism was positively associated with hospitalization-related NSSI, and neuroticism and neuroticism-related coping strategies were independent predictors of the risk of post-hospital NSSI. In summary, patients with NSSI show higher levels of neuroticism and lower levels of extraversion. However, there are few studies focusing on the personality characteristics of depression with NSSI. Only one study, Kang et al. (2021) found that patients with depression and NSSI showed higher levels of neuroticism and EPQ-psychoticism than patients with depression without NSSI. The study also found that, in addition to multiple mediating effects on NSSI, EPQ-psychoticism also has a direct impact on NSSI. Therefore, the personality characteristics of depression with NSSI are still worthy of further study.

In addition to neuroticism, EPQ-psychoticism, and extraversion, the defense style is also a crucial psychological factor for NSSI. Defense styles are automatic psychological processes that protect individuals from anxiety and awareness of internal or external danger (Association AP, 2000). Based on previous studies, defense styles can be classified as mature, intermediate, and immature types. Mature defense styles (e.g., anticipation, humor, sublimation, and inhibition) indicate an ability to cope well with external and internal conflict. Immature defense styles (e.g., projection, passive-aggression, performance, isolation, devaluation, autistic fantasy, denial, displacement, dissociation, splitting, rationalization, and somatization) indicate maladaptation to internal and external conflict and are more associated with severe psychopathology (e.g., depression, schizophrenia, eating disorders, etc.; Nishimura, 1998; Bond, 2004). Evans et al. (2005) found that, when facing difficulties, adolescents with NSSI showed less attention to problems and more avoidance behaviors in coping strategies than non-self-injurious adolescents, which seems to suggest that this kind of population adopts more immature defense and coping mechanisms in response to stress. In a study of non-clinical individuals, Sarno et al. (2010) found that both frequent and occasional NSSI individuals use more non-adaptive defense styles (projection, dissociation, fantasy, etc.) than non-self-injurious individuals, but there were no differences in adaptive defense styles. Brody and Carson (2012) also found that adolescents patient with NSSI presented more immature defenses and less mature defenses, with more immature defenses can predict NSSI. However, there are no studies on the defense styles of depression with NSSI.

Apart from a variety of psychiatric disorders as mentioned above, studies have found that NSSI is very common in individuals with personality disorders, particularly borderline personality disorder (BPD; Haw et al., 2001; Krysinska et al., 2006). In addition, NSSI seems to predict the development of BPD. Nakar et al. (2016) found a significant longitudinal association between NSSI and later diagnosis of BPD and borderline symptoms. Groschwitz et al. (2015) found that early-onset NSSI and prolonged NSSI in adolescents are predictors of adult BPD; however, some studies found no correlation between NSSI and BPD, suggesting other factor(s) may contribute to its association (Homan et al., 2017). In the context of depression which is associated with NSSI and BPD, investigation of the relationship between NSSI and BPD, especially for borderline personality trait that does not meet BPD diagnostic criteria is encouraged.

In conclusion, although many previous studies have focused on the personality traits, defensive styles, and borderline personality traits in NSSI individuals and patients with depression, there is only one study focused on the personality traits in patients with MDD and NSSI, and that study did not include a healthy control group and excluded a diagnosis of comorbid personality disorder. On the other hand, there are no studies focusing on defensive styles and borderline symptoms in patients with MDD and NSSI. There is a longstanding view that personalities represent a psychological structure that stabilizes over time and considers individuals’ personality traits can remain relatively constant over a considerable period of time (Dubois et al., 2020). However, previous studies have found that the personality of patients with depression will change as the illness continues and as treatments progress (Mullen et al., 1999; Bond, 2004). Based on this, we hypothesized that patients with MDD and NSSI present more prominent psychoticism, introversion, borderline symptoms, and immature defensive styles than patients with MDD without NSSI. Therefore, the purpose of this study is to compare whether there are differences in personality traits, defense styles, and borderline symptoms between patients with the first depressive episode with and without NSSI, and further explore the relationship between NSSI and these psychological indicators.

## 2. Materials and methods

### 2.1. Participants

This was a cross-sectional study on the major depressive disorder (MDD) and non-suicidal self-injury (NSSI). A total of 188 participants were recruited, including 124 patients with depression, from inpatients or outpatients in the Department of Depressive Disorders of Shenzhen Kangning Hospital from March 2020 to March 2022. The inclusion criteria were: (1) Meet the DSM-5 diagnosis of the first depressive episode (diagnosed by an experienced psychiatrist with the title of attending physician or above); (2) Age 18–35 years old; (3) Education level: junior high school and above. Exclusion criteria were: (1) Comorbid personality disorder using Structured Clinical Interview for DSM-IV Axis II Personality Disorders (SCID-II); (2) Severe physical illness or cerebral organic disease; (3) Comorbid psychiatric disorders other than NSSI, such as schizophrenia, bipolar disorder, anxiety disorders, etc. Of these 124 patients with depression, 60 of them had never experienced any NSSI in their lifetime (Major Depressive Disorder without Non-suicidal self-injury, MDD without NSSI group), and the other 64 patients with depression met the diagnostic criteria for NSSI in DSM-5 Section III Conditions Further Study (Major Depressive Disorder with Non-suicidal self-injury, MDD with NSSI group). NSSI was defined as the individual engaged in intentional self-inflicted damage to the surface of his or her body of a sort likely to induce bleeding, bruising, or pain (e.g., cutting, burning, stabbing, hitting, excessive rubbing; American Psychiatric Association D, Association AP, 2013). The rest of the 64 participants were healthy control subjects and were put in the healthy control group (HC group), who were doctors, nurses, interns from Shenzhen Kangning Hospital, and students from Shenzhen Yantian Senior High School. The inclusion criteria were: (1) Never had any NSSI in their lifetime; (2) Age 18–35; (3) Education level: junior high school and above. Exclusion criteria: (1) Diagnosed with any psychiatric disorder and personality disorder (using SCID-II), (2) combined with severe physical illness or cerebral organic disease. (3) The score of Beck Depression Inventory-II (BDI-II) ≥ 14. This study was reviewed and approved by the Ethics Committee of Shenzhen Kangning Hospital, and all participants gave informed consent to this study, and the informed consent was signed by the participants themselves.

### 2.2. Clinical assessment

#### 2.2.1. Demographic questionnaire

A self-administered demographic questionnaire, including name, gender, age, years of education, marital status, and lifestyle, was used to investigate the demographic information of the participants.

#### 2.2.2. Eysenck personality questionnaire (EPQ)

The Eysenck personality questionnaire (EPQ) was used to assess the participants’ personality factors. This study used the adult version of the Chinese version of the EPQ revised by Gong (1984). The scale contains 88 questions divided into four main factors: psychoticism, neuroticism, extraversion, and lie. The researchers used *T*-scores based on standardized data from the Chinese population—the higher the score of each factor, the higher the level of that personality trait of the individual. Although there are updated validated measures, this study used EPQ because EPQ has been introduced to China for a long time, and it is validated and widely used in the Chinese population. It is also recognized in the personality assessment of depression and has good reliability and validity. The Cronbach’s *α* coefficient of the four factors is 0.62–0.83, and the test–retest reliability is 0.63–0.87. In this study, all factor scores of EPQ were analyzed as continuous variables.

#### 2.2.3. Defensive style questionnaire (DSQ)

This study used the Defensive Style Questionnaire (DSQ) revised by Andrews et al. (1989) to assess participants’ defense styles. DSQ contains 88 items. The questionnaire scale includes three types of defensive styles: maturity (anticipation, humor, sublimation, and inhibition), intermediate (pseudoaltruism, idealization, and reaction formation), and immaturity (projection, denial, displacement, dissociation, and splitting,). The defensive style scores used in the current study consisted of the mean of the defense styles represented by each subscale [each item is rated on a 9-point Likert scale (1 = strongly disagree and 9 = strongly agree), with scores closer to nine indicating more frequent use of that defense style]. The questionnaire was translated into a Chinese version in 1993, and studies suggest that it has good reliability and validity for Chinese populations. The Cronbach’s *α* coefficient of the four factors is 0.61–0.88, and the test–retest reliability is 0.72–0.81. In this study, all factor scores of DSQ were analyzed as continuous variables.

#### 2.2.4. Ottawa self-injury scale (OSI)

The Ottawa self-injury scale (OSI) is a valid and reliable assessment tool for assessing the frequency and associated factors of NSSI in patients with NSSI (Nixon et al., 2015), with good reliability and validity as revised by the Chinese version (Wang et al., 2020). The OSI has a total of 28 items, and we used two of the questions from the first item (Have you committed non-suicidal self-injury? How many times have you self-injured in the past month?) to evaluate whether the participants had NSSI and the frequency of NSSI in the past month. The frequency of NSSI is divided into 4 levels: 0 (no NSSI in the last month), 1 (taking NSSI once in the last month), 2 (taking NSSI every week), and 3 (taking NSSI every day). The test–retest reliability of NSSI ideation and behavior frequency is 0.81 and the Cronbach’s α coefficient is 0.95. In this study, the frequency of NSSI was used as a rank categorical variable for correlation analysis and regression analysis.

#### 2.2.5. Beck depression inventory (BDI)

The study used Beck Depression Inventory (BDI) to assess depression severity. BDI is a commonly used and valid self-report measure of depressive symptoms. Its Chinese version has been widely used to assess depressive symptoms and severity in patients with psychiatric disorders and in the general population (Wang et al., 2020). This scale consists of 21 items, with a Likert scale of 4 (0–3), from 0 = no symptoms to 3 = severe symptoms and almost unbearable. The total scores range from 0 to 63, which reflects the overall severity of depressive symptoms. A total score of 14 or more indicates depression. The higher the total score, the more severe the depression. The Cronbach’s α coefficient of BDI in patients with MDD is 0.94, and the test–retest reliability is 0.55. The scores of BDI are continuous variables.

#### 2.2.6. Borderline symptom list (BSL)

The short version of the Borderline Symptom List (BSL) was used to assess borderline symptoms. BSL is a valid scale to rapidly assess symptoms and severity of borderline personality disorder, and this test has been translated into Chinese and evaluated for reliability and validity (Yang et al., 2018). The BSL consists of 23 items, each of which can be scored from 0 to 4 depending on severity. The total score is 92, with higher scores indicating more severe borderline symptoms. The Cronbach’s α coefficient for the BSL is 0.935–0.969. The scores of BSL are continuous variables.

### 2.3. Statistical analysis

The Kolmogorov–Smirnov single sample test was used to test whether the psychological indicators of each group were in a normal distribution. The results of the Kolmogorov–Smirnov single sample test showed that age (*p* = 0.151), years of education (*p* = 0.181), and the scores of extraversion (*p* = 0.078), EPQ-psychoticism (*p* = 121), and mature defense styles (*p* = 0.096) of the three groups were all normal distribution and the scores of neuroticism (*p* < 0.001), immature defense styles (*p* < 0.001), intermediate defense styles (*p* < 0.001), BDI (*p* < 0.001), and BSL (*p* < 0.001) were non-normally distributed. Demographic and clinical variables were compared between MDD with NSSI group, MDD without NSSI group, and HC group. The Chi-square test was used for categorical variables, analysis of variance (ANOVA) for continuous normally distributed variables, and pairwise comparisons using LSD. Non-normal distribution or variables with unequal variances were tested using the Wilcoxon rank-sum test and pairwise comparisons were performed using Kruskal–Wallis *H*-test. In addition, Pearson and Spearman correlations analysis, respectively, were used to evaluate the relationship between variables according to whether the variables fit a normal distribution. Finally, univariate logistic regression analysis was conducted among all patients with depression as a sample, NSSI as the dependent variable and each psychological indicator as independent variables one by one. A further multivariate logistic regression analysis was fitted by including all independent variables (forward LR method) to explore which ones were finally significantly associated with NSSI. All Data were analyzed using SPSS 26.0 statistical software and an alpha level of 0.05 for two tails was considered statistically significant.

*Post hoc* power analysis: based on the ANOVA model comparing the differences in EPQ subscore, DSQ subscore, and BSL score among groups, the effect size (*Cohen’s d*) ranged from 0.5 (intermediate defense style in DSQ score) to 1.9 (BSL score). Thus, the statistical power ranged from 0.87 to 1.00 by using the mean and standard deviation of these scores given the current sample size and type I error of 0.05.

## 3. Results

### 3.1. Demographic characteristics

A total of 220 subjects were invited to this study. Among them, 32 were excluded, including 19 in remission of depression with BDI < 14, 6 with comorbid anxiety disorder, and 7 unable to complete the scale assessment. There was no statistical difference in age and gender between the included and excluded groups. There were 188 participants included in this study finally, including 64 participants in the major depressive disorder (MDD) with non-suicidal self-injury (NSSI) group (47 females, mean age: 22.47 ± 3.63 years, range: 18–32 years), 60 participants in the MDD without NSSI group (43 females, mean age: 22.53 ± 3.86 years, range: 18–32 years), and 64 participants in the HC group (46 females, mean age: 22.83 ± 3.97 years, range: 18–32 years). There was no significant difference in age, gender, marital status, years of education, and residence status among these three groups of participants (all *p* < 0.05, see Table 1).

**Table 1:** Demographic, clinical characteristics of subjects and the frequency of non-suicidal self-injury (NSSI) last month in depressive disorder patients (MDD) with NSSI group.

	MDD with NSSI (*n* = 64)	MDD without NSSI (*n* = 60)	HC (*n* = 64)	*F*	*p*
Age, mean (SD)	22.47(3.63)	22.53(3.86)	22.83(3.97)	0.160	0.852
Range	(18, 32)	(18, 32)	(18, 32)		
Sex				0.059	0.971
Female, *n* (%)	47(73.4)	43(71.7)	46(71.9)		
Male, *n* (%)	17(26.6)	17(28.3)	18(28.1)		
Marriage				0.062	0.969
Married, *n* (%)	10(15.6)	9(15.0)	9(14.1)		
Unmarried, *n* (%)	54(84.4)	51(75.0)	55(85.9)		
Education years, mean (SD)	13.27(2.35)	12.88(2.48)	13.72(1.82)	2.307	0.102
Range	(9, 23)	(9, 20)	(10, 18)		
Living style				1.083	0.582
Living as single, *n* (%)	21(32.8)	19(31.7)	16(25)		
Living with others, *n* (%)	43(67.2)	51(68.3)	48(75)		
FON					
FON = 0, *n* (%)	20(31.3)	–	–		
1 ≤ FON < 1/week, *n* (%)	26(40.6)	–	–		
1/week ≤ FON < 1/day, *n* (%)	12(18.8)	–	–		
FON ≥ 1/day, *n* (%)	6(9.4)	–	–		

NSSI: non-suicidal self-injury; MDD with NSSI: major depressive disorder with non-suicidal self-injury; MDD without NSSI: major depressive disorder without non-suicidal self-injury; HC: healthy control subjects. FON: frequency of NSSI last month.

### 3.2. Differences in personality characteristics among the three groups

The results of the Chi-square test indicated that there are statistical differences in neuroticism (*z* = 56.482, *p* < 0.001) among the three groups. The results of the ANOVA indicated that there are statistical differences in extraversion (*F* = 9.407, *p* < 0.001, effect size = 0.114) and EPQ-psychoticism (*F* = 16.438, *p* < 0.001, effect size = 0.151) among the three groups. Patients with MDD and NSSI (68.39 ± 6.71, *p* < 0.001) and patients with MDD without NSSI (64.20 ± 8.05, *p* < 0.001) showed high levels of neuroticism than HC (47.81 ± 16.01), and there was no statistical difference in neuroticism levels between patients with MDD and NSSI and patients with MDD without NSSI (*p* = 0.072). Patients with MDD and NSSI (42.86 ± 13.91, *p* < 0.001) and patients with MDD without NSSI (47.23 ± 10.56, *p* < 0.006) showed lower levels of extroversion than HC (52.66 ± 13.54), while patients with MDD and NSSI showed lower levels of extroversion than patients with MDD without NSSI (*p* = 0.048). Patients with MDD and NSSI (56.55 ± 10.48) showed higher levels of EPQ-psychoticism than patients with MDD without NSSI (46.43 ± 9.13, *p* < 0.001) and HC (50.08 ± 10.22, *p* < 0.001), while patients with MDD without NSSI showed lower levels of EPQ-psychoticism than HC (*p* < 0.043; Table 2).

**Table 2:** Psychological measurements between MDD with NSSI group, MDD without NSSI group and HC group.

	MDD with NSSI (*n* = 64) mean (SD) [1]	MDD without NSSI (*n* = 60) mean (SD) [2]	HC (*n* = 64) mean (SD) [3]	*F*/*Z*	LSD/KWH
EPQ					
N	68.39 (6.71)	64.20 (8.05)	47.81 (16.01)	*Z* = 56.482***	[1] = [2] > [3]
E	42.86 (13.91)	47.23 (10.56)	52.66 (13.54)	*F* = 9.407***	[3] > [2] > [1]
P	56.55 (10.48)	46.43 (9.13)	50.08 (10.22)	*F* = 16.438***	[1] > [3] > [2]
L	47.73 (8.7)	47.08 (9.26)	44.92 (10.29)	*Z* = 1.927	[1] = [2] = [3]
DSQ					
Ma	4.94 (1.18)	5.00 (1.05)	5.22 (1.34)	*F* = 0.994	[1] = [2] = [3]
Im	5.35 (1.11)	4.60 (0.91)	3.79 (1.50)	*Z* = 41.393***	[1] > [2] > [3]
In	4.84 (0.74)	4.72 (0.61)	4.38 (1.29)	*Z* = 6.783*	[1]=[2], [2]=[3]
					[1] > [3]
L	4.47 (0.95)	4.61 (0.68)	4.59 (1.44)	*Z* = 1.726	[1] = [2] = [3]
BDI	33.22 (9.31)	24.42 (8.19)	3.95 (3.71)	*Z* = 136.709***	[1] > [2] > [3]
BSL	51.61 (22.05)	33.32 (18.14)	9.75 (8.73)	*Z* = 100.145***	[1] > [2] > [3]

NSSI: non-suicidal self-injury; MDD with NSSI: major depressive disorder with non-suicidal self-injury; MDD without NSSI: major depressive disorder without non-suicidal self-injury; HC: healthy control subjects. EPQ: Eysenck personality questionnaire; N: neuroticism; E: extraversion; P: psychoticism; L: lie; DSQ: defensive style questionnaire; Ma: mature defense style; Im: immature defense style; In: intermediate defense style; BDI: Beck depression scale; BSL: borderline symptoms list; SD: standard deviation; KWH: Kruskal–Wallis *H*-test.

^*^p < 0.05, ^**^p < 0.01. ^***^p < 0.001.

### 3.3. Differences in defense styles among the three groups

The results of the ANOVA indicated that there was no statistical difference in the mature defense styles among the MDD with NSSI (4.94 ± 1.18), MDD without NSSI (5.00 ± 1.05), and HC groups (5.22 ± 1.34; *F* = 0.994, *p* = 0.372). The results of the Chi-square test indicated that these three groups had statistical differences in immature defense styles (*z* = 41.393, *p* < 0.001) and intermediate defense styles (*z* = 6.783, *p* = 0.034). Patients with MDD and NSSI (5.35 ± 1.11, *p* < 0.001) and patients with MDD without NSSI (4.60 ± 0.91, *p* = 0.017) showed more immature defenses than HC (3.79 ± 1.50), while Patients with MDD and NSSI showed more immature defenses than patients with MDD without NSSI (*p* = 0.001). Patients with MDD and NSSI (4.84 ± 0.74) showed more intermediate defense styles than HC (4.38 ± 1.29, *p* = 0.034). There was no statistical difference in intermediate defense styles between patients with MDD and NSSI (4.84 ± 0.74) and patients with MDD without NSSI (4.72 ± 0.61, *p* = 1.000), and there was no statistical difference in intermediate defense styles between patients with MDD without NSSI (4.72 ± 0.61) and HC (4.38 ± 1.29, *p* = 0.221; Table 2).

### 3.4. Differences in depressive symptoms and borderline symptoms among the three groups

The results of the Chi-square test indicated that there were statistical differences in depressive symptoms (*z* = 136.709, *p* < 0.001) and borderline symptoms (*z* = 100.145, *p* < 0.001) among the MDD with NSSI, MDD without NSSI, and HC groups. MDD with NSSI (33.22 ± 9.31, *p* < 0.001) and MDD without NSSI groups (24.42 ± 8.19, *p* < 0.001) presented more depressive symptoms than HC (3.95 ± 3.71), while patients with MDD and NSSI had more depressive symptoms than patients with MDD without NSSI (*p* = 0.003). Similar findings: the MDD with NSSI (51.61 ± 22.05, *p* < 0.001) and MDD without NSSI groups (33.32 ± 18.14, *p* < 0.001) presented more borderline symptoms than the HC group (9.75 ± 8.73), and the MDD with NSSI group had more borderline symptoms than the MDD without NSSI group (*p* = 0.001; Table 2).

### 3.5. The relationship between NSSI and the psychological factors

Spearman correlations analysis (Table 3) showed that in the MDD with NSSI group, NSSI frequency in the last month was negatively correlated with mature defense styles (*r* = −0.293, *p* = 0.019) and positively correlated with depressive symptoms (*r* = 0.446, *p* < 0.001) and borderline symptoms (*r* = 0.430, *p* < 0.001). There were no significant correlation between NSSI frequency in the last month and neuroticism, extroversion, EPQ-psychoticism, immature defense styles, and intermediate defense styles (all *p* > 0.05). Regression analysis Model 1 was used to investigate the correlations between NSSI and each psychological indicator by univariate regression analysis. The results showed correlations between NSSI and immature defense styles, EPQ-psychoticism, neuroticism, depressive symptoms, and borderline symptoms (all *p* < 0.05). The results of multivariate logistic regression analysis show that EPQ-psychoticism (OR = 1.097, *p* < 0.001) and depressive symptoms (OR = 1.098, *p* < 0.001) were independent risk factors of taking NSSI in patients with depression (Table 4).

**Table 3:** The relationship between the frequency of NSSI last month and psychological measurements in MDD with NSSI group.

	*r*	*p*
N	0.117	0.359
E	−0.023	0.856
P	0.024	0.850
Ma	−0.293	0.019
Im	0.080	0.532
In	0.115	0.365
BDI	0.446	0.000
BSL	0.430	0.000

NSSI: non-suicidal self-injury; MDD with NSSI: major depressive disorder with non-suicidal self-injury; N: neuroticism; E: extraversion; P: psychoticism; Ma: mature defense style; Im: immature defense style; In: intermediate defense style; BDI: Beck depression scale; BSL: borderline symptoms list.

**Table 4:** Results of univaraiate and multivariate logisctic regression analysis for NSSI.

	Univariate logistic regression		Multivariate logistic regression	
	OR (95% CI)	*p*	OR (95% CI)	*sp*
N	1.082 (1.026, 1.140)	0.003	NS	–
E	0.972 (0.943, 1.001)	0.055	NS	–
P	1.110 (1.062, 1.161)	<0.001	1.097 (1.047, 1.150)	<0.001
Ma	0.954 (0.695, 1.310)	0.773	NS	–
Im	2.905 (1.412, 3.109)	<0.001	NS	–
In	1.294 (0.760, 2.204)	0.342	NS	–
BDI	1.115 (1.065, 1.168)	<0.001	1.098 (1.046, 1.151)	<0.001
BSL	1.044 (1.024, 1.065)	<0.001	NS	–

NSSI status (yes vs. no) was set as independent variable in both Logistic regression models. Univariate logistic regression analysis was performed to investigate the correlation of each psychological indicator with NSSI. Multivariate logistic regression analysis was performed including all the variables using forward LR method (*p* < 0.05). NSSI: non-suicidal self-injury; N: neuroticism; E: extraversion; P: psychoticism; Ma: mature defense style; Im: immature defense style; In: intermediate defense style; BDI: Beck depression scale; BSL: borderline symptoms list. OR: odds ratio; CI: confidence interval; NS: not significant.

## 4. Discussion

To the best of our knowledge, this is the first study to address the personality traits, defensive styles, and borderline symptoms in first-episode depressive disorder. We excluded the possibility of comorbid borderline personality disorder in patients and set up a healthy control group (HC).

### 4.1. Personality and NSSI

Our study found that, compared with HC, both patients with major depressive disorder (MDD) and non-suicidal self-injury (NSSI) and patients with MDD without NSSI presented higher levels of neuroticism and lower levels of extraversion, which is consistent with Carvalho et al. (Grace and O'Brien, 2003). As a very important personality trait, neuroticism is associated with negative emotions (depression, anxiety, tension, guilt, shame, etc.; Eysenck and Eysenck, 1975) and emotional vulnerability (moodiness, etc.; Lahey, 2009). The results of our study suggest that patients with depression show more significant negative emotions and emotional instability than HC. Extroversion is characterized by being carefree, sociable, vivacious, confident, etc. (Eysenck and Eysenck, 1985). In this study, patients with MDD and NSSI showed lower levels of extroversion than patients with MDD without NSSI, suggesting that patients with MDD and NSSI showed more pronounced personality traits such as anxiety, emptiness, inferiority, and poor communication characteristics than patients with MDD without NSSI and HC. NSSI can be triggered by adverse interpersonal conflicts (Hepp et al., 2021), suggesting that introversion may be a personalistic risk factor for depression patients to engage in NSSI.

This study found that patients with MDD and NSSI presented higher levels of EPQ-psychoticism than patients with MDD without NSSI, which is consistent with the findings of a recent study (Kang et al., 2021). It is worth mentioning that our study excluded the effects of recurrent depressive episodes and comorbid borderline personality disorder on the personalities of patients with depression. Interestingly, our study found that patients with MDD and NSSI showed higher levels of EPQ-psychoticism than HC, while patients with MDD without NSSI had lower levels of EPQ-psychoticism than HC. A study did not find differences in the level of EPQ-psychoticism between depression and healthy controls (Grace and O'Brien, 2003), which may be because the study did not further differentiate between depressed patients with and without NSSI, and NSSI may have acted as a confounding variable to influence the results consistency. Additionally, in this study, regression analysis also found that EPQ-psychoticism is an independent risk factor for NSSI in patients with depression. A recent study suggested that EPQ-psychoticism has both direct and indirect effects on NSSI through the structural equation modeling (Kang et al., 2021). These results indicate that EPQ-psychoticism may be a predictor of NSSI in patients with depression. In the Eysenck scale, psychoticism represents egocentricity, apathy, stubbornness, obstinacy, impulsiveness, hostility, aggressiveness, suspicion, etc. (Eysenck and Eysenck, 1975), and our study suggests that patients with depression with these personality traits are more likely to engage in NSSI. This is consistent with the findings of some previous studies, which also found that patients with NSSI showed more interpersonal problems (Turner et al., 2016), impulsivity (Peters et al., 2016), and aggression (Terzi et al., 2017).

### 4.2. Defense styles and NSSI

Defense styles are used to describe the ways in which individuals use to buffer internal conflicts. Defense styles can be classified according to rank from high levels to low levels as mature defense styles, intermediate defense styles, and immature defense styles, with immature defense styles being the most closely related to the severity of psychopathology (Bond, 2004). Our study found that both patients with MDD and NSSI and patients with MDD without NSSI presented more immature defense styles than HC, which is consistent with several previous studies (Corruble et al., 2004; Bronnec et al., 2005; Blaya et al., 2006; Calati et al., 2010), suggesting that patients with depression may adopt more non-adaptive coping strategies when facing internal conflicts. This study also found that depression with NSSI presented more immature defenses than depression without NSSI, which further indicated that these non-adaptive ways of coping with inner conflict were more prominent in the group of patients with MDD and NSSI. Several previous studies mentioned above have also found that patients with depression presented more intermediate defense styles than healthy populations. Our study replicates this finding but extends it by showing the difference exists only between patients with MDD and NSSI and HC, whereas there is no significant difference in intermediate defense styles between patients with MDD and NSSI and HC. Previous studies have also found that NSSI individuals use intermediate defense styles more than non-self-injurious individuals (Sarno et al., 2010; Brody and Carson, 2012). This seems to suggest that in patients with MDD and NSSI, NSSI is more closely related to intermediate defense styles than the depression itself. Studies have found that immature defense styles and intermediate defense styles are predictors of poor interpersonal relationships (Joyce et al., 2013) and negative emotions (Carvalho et al., 2013), and these factors are the main reasons for individuals to engage in NSSI (American Psychiatric Association D, Association AP, 2013). This suggests that immature and intermediate defense styles may be NSSI mediated by bad interpersonal relationships and negative emotions, but the findings need further investigation. The correlation analysis in this study found that NSSI frequency in the past month was negatively correlated with the mature defense styles, which are adaptive ways for individuals to cope with inner conflicts and reflects their good psychological functioning. Our study suggests that mature defense styles may be a protective factor against NSSI.

### 4.3. Depression, borderline symptoms and NSSI

In our study, patients with MDD and NSSI and patients with MDD without NSSI had higher depressive symptom scores than HC, and patients with MDD and NSSI had severer depression than patients with MDD without NSSI. This study also found that the frequency of NSSI in the past month was positively correlated and depression severity. Additionally, further regression analysis suggested that depression severity was a risk factor for NSSI in patients with depression, supporting the findings of previous research (Plener et al., 2015), which suggests that depressive symptoms are an independent predictor of patients’ NSSI. Depression, as one of the common negative emotions, may cause intense emotional stress to patients, and NSSI can be an effective way to relieve the emotional stress (Tilton-Weaver et al., 2019). The rate of comorbidity between depression and borderline personality disorder is very high, and previous studies have found that the two disorders have many commonalities in phenomenological and pathological mechanisms (Galione and Oltmanns, 2013; Nenov-Matt et al., 2020; De la Pena-Arteaga et al., 2021; Villarreal et al., 2021).

This study found that patients with MDD and NSSI and patients with MDD without NSSI presented more borderline symptoms than HC. A study also found that patients with persistent depressive disorder had significantly higher borderline personality symptom scores than healthy control subjects (Nenov-Matt et al., 2020), which is consistent with our findings. This study also found that patients with MDD and NSSI presented more borderline symptoms than patients with MDD without NSSI, suggesting that patients with MDD and NSSI may show more borderline personality traits compared to patients with MDD without NSSI. In the MDD with NSSI group, the frequency of NSSI in the past month was positively correlated with depressive symptoms and borderline symptoms. Regression analysis suggested that depressive symptom was an independent risk factor for predicting NSSI in patients with depression, further demonstrating that the high degree of intertwined experiences of borderline symptoms and depressive symptoms may contribute to the development and exacerbation of NSSI. Our study found that patients with MDD and NSSI presented more borderline personality than patients with MDD without NSSI. Numerous studies found that compared with patients with single depression, patients with depression comorbid with other psychiatric disorders responded less well to antidepressant treatment medication, and comorbidity often affects the treatment effect of depression (Mogi and Yoshino, 2017; Gronemann et al., 2020). This clinical phenomenon is also seen in patients with MDD and NSSI (Asarnow et al., 2011). Earlier studies have found that treatment of comorbidities can also effectively improve depressive symptoms in patients with depression (Gunderson et al., 2004). This finding provides an idea for clinical work: those effective approaches for the treatment of borderline personality disorder [dialectical behavior therapy (Linehan et al., 2015) and mentalization-based treatment (Beck et al., 2020)] may be able to help patients with MDD and NSSI who neither meet the diagnostic criteria for borderline personality disorder nor respond well to antidepressant therapy, and this is supported by a number of clinical studies (Rossouw and Fonagy, 2012; Mehlum et al., 2014).

This study has the following advantages. Firstly, we recruited first-episode patients with depression to eliminate the influences of disease duration and long-term treatment on relevant psychological indicators in this study. Secondly, we recruited the healthy control subjects as the control group, and the sample sizes were largely matched among the MDD with NSSI group, MDD without NSSI group, and HC group. There were no statistical differences in demographic characteristics, such as age and gender among the three groups. In addition, we paid special attention to borderline symptoms, a psychological indicator closely associated with NSSI. However, the study has the following limitations. Firstly, the study only focused on patients with depression, so it is unclear whether the findings of the personality characteristics, defensive styles, and characteristics of borderline symptoms in patients with NSSI are applicable to other psychiatric disorders. Secondly, the samples of this study were collected during the COVID-19 pandemic. Although the outbreak of COVID-19 has been effectively controlled since early 2020 and Shenzhen is relatively less affected by the pandemic, it is still important to consider the impact of the pandemic on the onset of depression. In this study, all participants were not confined at home during the pandemic. Thus, we expected the influence would be limited. Thirdly, though the majority of participants in the healthy control group were medical students and high school students, there was a small sample size of doctors and nurses in the healthy control group, which may be motivated to underreport and cause biased results. Future studies need to consider this problem and avoid the probability of underreporting. Fourthly, we adopted BSL as a tool to capture borderline personality traits with a restricted range of scores as we excluded participants with BPD in this study. However, we excluded BPD in this study aiming to exclude the confounding effect of BPD. Fifthly, Fifthly, the EPQ provides only standardized scores with the intention of reducing the effect of age and gender on the assessment results. Future research should replicate these results with other personality measures that examine the same personality traits, as the standardization of the scores may have affected the results. Sixthly, we analyzed the data several times separately in the regression model, potentially increased type I error. Future studies are needed to replicate these findings to make them more solid (page 14). Finally, this study was conducted as a cross-sectional study, so causal inferences about the results cannot be made. Future longitudinal studies will help to further elucidate the relationship between NSSI, borderline symptoms and personality traits, and defense styles.

In summary, the current study found that patients with MDD and NSSI presented more neuroticism, introversion, EPQ-psychoticism, immature defense styles, depressive symptoms, and borderline symptoms than patients with MDD without NSSI and HC, suggesting that these indicators may be involved as psychological factors in the pathogenesis of NSSI in patients with depression.

## Data availability statement

The raw data supporting the conclusions of this article will be made available by the authors, without undue reservation.

## Ethics statement

The Ethics Committee of Shenzhen Kangning Hospital approved the research protocol. All patients/participants provided their written informed consent to participate in this study.

## Author contributions

BP for paper design, data analysis, writing, and revision. YL for data collection and writing. GJ, JY, ZW, JZ, YY, XL, and YW for data collection. JL for paper revision. JP and YZ for paper supervision. All authors have read and agreed to the final text.

## Funding

This study was supported by the Shenzhen Key Medical Discipline Construction Fund (No. SZXK041) and the Shenzhen Fund for Guangdong Provincial High-level Clinical Key Specialties (No. SZGSP013). These sources had no further role in study design, data collection and analysis, report writing, and the decision to submit the paper for publication.

## Conflict of interest

The authors declare that the research was conducted in the absence of any commercial or financial relationships that could be construed as a potential conflict of interest.

## Publisher’s note

All claims expressed in this article are solely those of the authors and do not necessarily represent those of their affiliated organizations, or those of the publisher, the editors and the reviewers. Any product that may be evaluated in this article, or claim that may be made by its manufacturer, is not guaranteed or endorsed by the publisher.
